# Domestic violence and associated factors among pregnant women in sub-Saharan African countries from the recent demographic and health survey data: a multilevel analysis

**DOI:** 10.3389/fpubh.2024.1386524

**Published:** 2024-08-27

**Authors:** Mamaru Melkam, Setegn Fentahun, Gidey Rtbey, Fantahun Andualem, Girum Nakie, Techilo Tinsae, Yilkal Abebaw Wassie, Beminate Lemma Seifu, Bezawit Melak Fente

**Affiliations:** ^1^Department of Psychiatry, College of Medicine and Health Science, University of Gondar, Gondar, Ethiopia; ^2^Department of Medical Nursing, College of Medicine and Health Science, University of Gondar, Gondar, Ethiopia; ^3^Departments of Public Health, College of Medicine and Health Science, Samara University, Samara, Ethiopia; ^4^Department of General Midwifery, College of Medicine Health Science, School of Midwifery, University of Gondar, Gondar, Ethiopia

**Keywords:** multilevel, sub-Saharan Africa, intimate (heterosexual) relationships, partner, violence

## Abstract

**Introduction:**

Intimate partner violence (IPV) is a human rights violation that often involves violence against women, which appears to be the most prevalent type of abuse. IPV is a global public health issue with major human rights violations. Pregnant women’s IPV needs special consideration because of the possible harm that might happen to mothers and their fetuses. The enormous global public health issue of IPV affects physical, mental, and sexual transgressions. Even though there were studies conducted on IPV among women, few studies were conducted among pregnant women in sub-Saharan African countries. Therefore, this study revealed IPV and associated factors among pregnant women from the recent Demographic and Health Survey (DHS) in sub-Saharan African countries.

**Methods:**

Multilevel logistic regression analysis used data from the recent sub-Saharan African countries DHS was carried out using this secondary data. For this study, pregnant women between the ages of 15 and 49 were included; the total sample size was 17,672. Multilevel logistic regression models were calibrated to determine the associated factors at the individual and community level with IPV, with a 95% CI and AOR.

**Results:**

The prevalence of IPV among pregnant women in 23 sub-Saharan African countries was 41.94%, with a 95% CI of 40.82 to 43.06%. Poorer and poorest [AOR = 1.92; 95% CI: (1.01, 3.67)] and [AOR = 2.01; 95% CI:(1.02, 3.92)], partner alcohol drink [AOR = 3.37;95% CI:(2.21, 5.14)], and no partner education [AOR = 2.01;95% CI:(1.12, 3.63)] were statistically associated factors with IPV among pregnant women.

**Conclusion:**

The prevalence of IPV among pregnant women in sub-Saharan African countries was high (41.94%). Low economic status, partner drinking alcohol, and partner no education were the associated factors of IPV. This finding provides clues for policymakers and other organizations concerned about women.

## Introduction

Intimate Partner Violence (IPV) is defined by the World Health Organization (WHO) as the intentional actions of an intimate partner or former spouse that cause mental distress, physical injury, sexual abuse, or inherited behaviors (1). The international community is coming to recognize physical violence against women as a serious violation of human rights. The findings show that women in developing nations are more likely than those in developed countries to experience physical violence from intimate partners (1, 2). Physical violence affects women’s health in two ways: directly, when it results in injuries, and indirectly, when it causes chronic illnesses that can result from ongoing stress (3). Sexual, emotional, and physical abuse of women and girls undermines their wellbeing and means of subsistence and upends their social networks and interpersonal relationships (4).

IPV is a hazardous health issue for public health worldwide, with a higher frequency in low-income nations (4). Research revealed that women who have experienced sexual abuse frequently face emotional, physical, financial, and social repercussions, including substance abuse, despair, anxiety, and addiction to sexual activity (5). IPV has a negative effect, particularly among pregnant women; it is especially concerning for them. It could result in increased rates of sexually transmitted diseases (STDs), postpartum hemorrhage, anxiety, depression, and eating disorders, as well as numerous complications (such as placenta abruption, placenta previa, preeclampsia, gestational diabetes, and antepartum hemorrhage) (6–8).

IPV is a significant public health issue in the United States, where 36% of women would at some point in their lives encounter rape, physical abuse, or stalking at the hands of an intimate partner (4). There has been variation in the prevalence of IPV both nationally and locally, which evidences low levels of communal empowerment for women (9, 10). Furthermore, women who experienced violence both before and throughout their pregnancies reported experiencing higher levels of intensity and frequency of pregnancy-related violence (11). The prevalence of IPV is high in low- and middle-income countries. Global estimates of IPV against pregnant women range from 3 to 30% (12). In Nigeria and Kenya, the burden of pregnant women who had IPV was 33 and 37%, respectively (13, 14). The continent of Africa has the greatest rate of IPV during pregnancy. Between 2 and 57% of pregnant women in Africa experience IPV; a meta-analysis produced a pooled value of 15.23% (15). The burden of IPV in Ethiopia was 48, 39.9, and 28.74% (11, 16, 17).

No one element can entirely clarify why some people are violent or whether violence tends to be more common in some groups than others. Instead, there are a variety of underlying causes for IPV against women (18, 19). It has an impact on every aspect of women’s lives, including their capability to take care of themselves and their children, be productive, be autonomous, and engage in social activities (11). The most determinant factors associated with IPV among pregnant women in low- and middle-income countries showed considerable variation, including women’s empowerment, partner and respondent occupations, household sex composition, age gap between spouses, wealth index, and the education levels of both the respondent and her husband (11, 16, 17, 20). The other factors associated with IPV were incorporated, including partner alcohol drinking, distance from health facilities, residency, marital status, and mass media exposure (11, 17, 21, 22).

There is proof that evaluating different exposures to high-risk pregnant mothers needs to be a primary concern for the health of the pregnant mothers (20). There is not much research on how prenatal violence affects communities, even though violence differs depending on the community. The first step in developing and implementing strategies that mitigate and treat consequences is determining the prevalence of IPV during pregnancy. Even though there are studies conducted in different countries among pregnant mothers, the prevalence is inconsistent. Therefore, this study revealed the burden of IPV among pregnant mothers and its determinant factors in sub-Saharan Africa from the recent DHS.

## Methods

### Study area and setting

Recent DHS of sub-Saharan African countries was used for this multilevel analysis. Sub-Saharan African countries included in this study with their respective data collection year were Angola (2015–16), Benin (2017–18), Burundi (2016–17), Cameroon (2022), Chad (2014–15), Ethiopia (2016), Gambia (2019–20), Gehana (2022), Guinea (2018), Kenya (2022), Lesotho (2014), Liberia (2019–20), Malawi (2015–16), Mali (2018), Nigeria (2018), Rwanda (2019–20), Senegal (2019), Sierra Leone (2019), South Africa (2016), Tanzania (2022), Uganda (2016), Zambia (2018), and Zimbabwe (2015).

Data from the sub-Saharan African countries DHS include a wide range of objectives focusing on fertility, reproductive health, maternal and child health, mortality, nutrition, and self-reported health on self-reported behaviors among adults. The KDHS provided datasets on men, women, children, births, and households for the survey. The individual record dataset (IR file) consists of data extracted from this survey. In the first step, 1,417 enumeration area (EA) clusters were selected from the sub-Saharan African countries’ Household Health Survey framework using the equal probability selection method. Participants were pregnant women aged 15 to 49 from sub-Saharan African countries who served as source populations. The final weighted sample size for this secondary data analysis was 17,672 pregnant women from the 23 sub-Saharan African countries (DHS). Detailed information on the data is available on the official link, http://www.dhsprogram.com/ (23).

### Variables of the study

#### Dependent variables

IPV was measured by self-reported questionnaires, including physical, sexual, and emotional violence, which were considered the dependent variables of the study by the modified Conflict Tactic Scales of Straus (24). The IPV was measured by the following nine questions and having at least one form of violence was considered IPV.

##### Physical violence

Ever been kicked or dragged by your husband?

Ever been strangled or burned by a husband?

Ever been threatened with a knife, gun, or another weapon?

##### Sexual violence

Ever been physically forced to have unwanted sex by your husband?

Ever been forced to do other sexual acts by your husband?

Ever been forced to perform sexual acts that the respondent did not want to?

##### Emotional violence

Ever been humiliated by your husband?

Ever been threatened with harm by your husband?

Ever been insulted or made to feel bad by your husband?

#### Independent variables

Independent variables were extracted from the recent data of DHS of 23 sub-Saharan African countries, including household variables, wealth index, and reproductive-related variables. The variables that were extracted as independent variables for this study included age, sex of the household head, number of children, distance from the health facility, current marital status, religion, ethnicity, residence, occupation of respondent and partner, education level of respondent and partner, age difference between spouse, and husband/partner alcohol use. Community-level variables used for this study included place of residency (urban and rural), educational level (low and high), wealth index (low and high), and media exposure (low and high). The histogram was used to examine the distribution of the proportion values that were calculated for every community-level variable. Finally, mean and median values were used for dichotomic skewed and normally distributed variables, respectively.

### Data management and analysis

Data extraction, coding, cleaning, and analysis were conducted using Stata version 14 software. Frequency and percentage were among the descriptive statistics that were completed in a table and text. Using sample weight with cluster, the analysis’s non-proportionate allocation and representativeness of the sample were carried out. An analysis that was multilevel was carried out to preserve the collected data’s hierarchical structure. Multilevel bivariable logistic regression analysis was conducted to determine the associated variables to be entered into multivariable analysis with a *p*-value of less than 0.25. The statistically substantially associated variables with *p*-values of less than 0.05 were identified using multilevel multivariable logistic regression analysis, and an adjusted odd ratio (AOR) with a 95% confidence interval (CI) was computed.

Four model analyses were carried out for the multivariable, multilevel logistic regression study. The initial model, also known as the null model, was run without the use of any explanatory variables. Only the individual-level variables were fitted in the second model; community-level variables were included in the third model; and both individual and community-level variables were fitted in the fourth model. The models were compared and their fitness was evaluated using Deviance and the Akaike Information Criterion (AIC); the model that received the lowest score was considered to be the best fit. The degree of heterogeneity among intimate relationship violence between the clusters was also measured using the intra-class correlation (ICC); ICC = $\frac{VA}{VA+3.29}*100\%$ . (the percentage of the total observed individual variance in IPV that can be attributable to differences between clusters). To measure the variation in IPV among clusters, the median odds ratio (MOR) = e^0.95√VA^ was used (25). Both the measurement of the odd ratio scale variation of IPV in the cluster and the degree of homogeneity of the evaluation of IPV were conducted. Ultimately, factors statistically substantially related to IPV were identified with a *p*-value of less than 0.05, and the AOR with 95% CI was computed.

## Results

### Descriptive characteristics of respondents

A total of 17,672 pregnant women from the age of 15 to 49 were included in this secondary data analysis. Of the study participants, 46.19% were between the ages of 25 and 34, and 71.88% of the participants were male household-headed. Of the study participants, 38.45% were orthodox religion followers and 74.33% of the study participants were partner alcohol users. Of the study participants, 22.33% were the richest, and 64.75% of the study participants had mass media exposure. Of the study participants, 29.68% of the study participants had no child at the time of data collection. Of the study participants, 56.00% lived in urban areas as their place of residence. Of the study participants, 55.57% had 1–2 children and of the study participants, 41.60 were at a distance from their health facility (Table 1).

**Table 1:** Socio-demographic characteristics of study participants of sub-Saharan African countries (*n* = 17,672).

Variables	Category	Weighted frequency (*n* = 17.7)	Percentage (%)
Age	15–24	4.208	23.81
	25–34	8.163	46.19
	35–39	2.474	14.00
	40–49	2.827	16.00
Household sex composition	Female	4.970	28.12
	Male	12.702	71.88
Maternal educational	No education	14.850	84.03
	Primary	1.197	33.78
	Secondary	1.405	39.66
	Higher	220	6.21
Partner educational level	No education	15.390	87.09
	Primary	726	4.11
	Secondary	1.217	6.89
	Higher	339	1.92
Religion	Orthodox	6.795	38.45
	Catholic	3.932	22.25
	Protestant	2.662	15.06
	Muslim	1.142	6.46
	Other religions^*^	3.141	17.78
Partner occupation	Not working	4.183	23.67
	Working	13.489	76.33
Partner alcohol use	Yes	13.135	74.33
	No	4.537	25.67
Distance of health facility	Yes	7.352	41.60
	No	10.320	58.40
Partner occupation	Not working	5.312	30.06
	Working	12.360	69.94
Media exposure	Yes	11.443	64.75
	No	6.229	35.25
Wealth status	Poorest	3.692	20.89
	Poorer	3.250	18.39
	Middle	3.220	18.22
	Richer	3.563	20.16
	Richest	3.947	22.33
Number of children	0	5.245	29.68
	1–2	9.820	55.57
	3–4	2.014	11.40
	≥5	593	3.36
Age gap	<5	5.229	29.59
	≥5	12.443	70.41
Community-level Wealth index	High	708	49.96
	Low	709	50.04
Community-level Media exposure	High	709	50.04
	Low	708	49.96
Community-level Educations	High	714	50.39
	Low	703	49.61
Residence	Urban	7.776	44.00
	Rural	9.896	56.00

*Other religion like atheist and traditionalist.

### Prevalence of IPV among pregnant women

The prevalence of IPV among pregnant women in 23 sub-Saharan African countries was 41.94%, with a 95% CI of 40.82 and 43.06%. Of this prevalence, 15.76% had physical violence, 35.88% had emotional violence, and 14.73% had sexual violence.

### Model fitness and statistical analysis

IPV within the cluster was associated with 1.73% of respondents’ changes in the ICC of the null model (model I). The MOR of IPV in the null model was 1.73, indicating that there was variation between the clusters. The odds of an individual experiencing IPV were 1.73 times higher in the cluster with a higher risk of these disorders than in the cluster with a lower risk, assuming a single participant was randomly picked from each of the two clusters. Model IV was the most well-fitting model for this investigation since it had the lowest AIC and deviation value (Table 2).

**Table 2:** Multilevel multivariable logistic regression of sub-Saharan African countries demographic and health survey data analysis (*n* = 17,672).

Variable	Null model	Model I	Model II	Model III
Household sex composition				
Male		1		1
Female		1.03 (0.90, 1.17)		1.03 (0.62, 1.72)
Age				
15–24		1		1
25–34		1.45 (1.22, 1.73)		1.65 (0.84, 3.22)
35–39		1.33 (1.14, 1.55)		1.43 (0.81, 2.52)
40–49		1.09 (0.90, 1.33)		1.68 (0.81, 3.47)
Wealth index				
Poorest		1.25 (1.06, 1.48)		**1.92 (1.01, 3.67)**
Poorer		1.49 (1.25, 1.77)		**2.01 (1.02, 3.92)**
Medium		1.28 (1.08, 1.52)		1.81 (0.37, 1.76)
Richer		1.15 (0.98, 1.36)		1.20 (0.66, 2.19)
Richest		1		1
Health facility distance				
Yes		1.24 (1.11, 1.38)		1.24 (0.80, 1.92)
No		1		1
Partner education				
No education		1.32 (1.14, 1.54)		**2.01 (1.12, 3.63)**
Primary		3.36 (2.10, 5.36)		1.86 (0.25, 2.90)
Secondary		2.04 (1.31, 3.18)		1.87 (0.57, 1.04)
Higher		1		1
Partner alcohol drink				
Yes		2.78 (2.50, 3.10)		**3.37 (2.21, 5.14**)
No		1		1
Media exposure				
No		1.04 (0.93, 1.18)		0.78 (0.49, 1.28)
Yes		1		1
Maternal occupation				
Working		1.30 (1.16, 1.46)		1.03 (0.67, 1.58)
Not working		1		1
Maternal education				
No education		1.15 (0.66, 2.00)		0.44 (0.13, 1.53)
Primary		2.26 (1.29, 3.94)		0.51 (0.13, 1.02)
Secondary		1.96 (1.13, 3.39)		0.86 (0.25, 2.90)
Higher		1		1
Partner occupation				
Working		3.49 (2.20, 5.53)		1.44 (0.13, 1.53)
Not working		1		1
Community-level variables				
Residence				
Urban			1	1
Rural			1.63 (1.13, 2.36)	1.53 (0.94, 2.50)
Media exposure				
High			1	1
Low			1.07 (0.74, 1.56)	1.02 (0.68, 1.53)
Education				
High			1	1
Low			1.23 (0.85, 1.79)	1.20 (0.80, 1.80)
Wealth status				
High			1	1
Low			1.40 (0.93, 2.08)	1.30 (0.83, 2.03)
Likelihood ratio	−4981.0819	−4685.4188	−358.92194	−324.79085
ICC	0.0918087	0.091284	0.0495375	0.0613919
Deviance	9962.16	9370.83	717.84	649.58
AIC	2315.789	2286.526	2155.27	2052.27
BIC	2248.387	2133.219	1827.62	1817.62
MOR	1.73			

AIC, Akaike Information Criterion; BIC, Bayesian Information Criteria; ICC, Intra-class correlation; MOR, Median odds ratio. The bold values are variably statistically significantly associated.

### Factors associated with intimate partner violence

In bivariable multilevel analysis, factors associated with IPV with a *p*-value of <0.25 were household sex, distance from health facilities, partner occupations, maternal education, partner education, wealth index, mass media exposure, maternal occupations, residence, age, and partner alcohol drink at the individual level and residence and wealth index were associated factors at the community level. In multivariable multilevel analysis, poorer and poorest status of wealth, no partner education, and partner alcohol drink were associated with a *p*-value of <0.05.

The odds of the occurrence of IPV were 1.92 and 2.01 times higher among the poorer and poorest as compared with the richest wealth status [AOR = 1.92; 95% CI: (1.01, 3.67)] and [AOR = 2.01; 95% CI:(1.02, 3.92)], respectively. The odds of IPV were 3.37 times higher among partners who have drunk alcohol as compared with those who do not drink alcohol [AOR = 3.37;95% CI:(2.21, 5.14)]. Participants with partners who have no education were 2.01 times more likely to have IPV as compared to others whose partners have a higher level of education [AOR = 2.01;95% CI:(1.12, 3.63); Table 2].

## Discussion

The prevalence of IPV among pregnant women was 41.94%, with a 95% CI of 40.82 and 43.06%. Multilevel analysis prevalence carried out in 23 sub-Saharan African countries DHS secondary data analysis was high. This study provided the burden and the factors related to IPV among pregnant women but it did not show the exact causal inference for IPV. The findings of this study are in line with other studies conducted in Ethiopia among pregnant women (26) 41.1%. The findings of this study are lower than those of other studies conducted in Ethiopia 59% (27). The probable reason for this discrepancy might be that the observed deviation from research conducted in Ethiopia may have resulted from cultural differences from other African countries included in this study (27). The sensitivity of disclosing IPV is a common cultural issue across all African countries, but it is particularly pronounced in Ethiopia due to its specific religious and cultural norms. Moreover, a variation in the sample size they used could be the likely reason for the variance since our study used a large sample size from sub-Saharan African countries.

In other ways, this finding is higher than other studies conducted on pregnant women from DHS data in Ethiopia 28.74% (11), and East Africa 37.14% (28). This is also higher than other studies conducted in Jefferson 7.4% (21). This discrepancy compared to other DHS data may be due to Ethiopian women not disclosing their experiences of violence, coupled with the cultural sensitivity surrounding IPV in Ethiopia (11). The other effect is that the attention given to gender equality leads to different types of violations (28). This discrepancy might be because Ethiopian society is extremely patriarchal; women frequently feel degraded and ashamed to report assault (mostly sexual violence) out of fear of social norms and other unfavorable reactions (11). The other reason could be the different sample sizes, cultural differences, and the effect of law on the violence variation that creates this discrepancy. The discrepancy may be attributed to variations in the implementation of rules and regulations, in addition to cultural differences. The laws of African countries were not similar in protecting the rights of women.

A low level of wealth index was one of the associated factors with IPV. This association is in concordance with other studies conducted in Jefferson (21) and Kenya (29, 30). The possible reason for this association could be that the effect of IPV may decrease as a result of initiatives to increase low-income communities’ accessibility to housing (21). When a feeling of being stuck and helpless to get out of these situations is combined with high levels of local disturbance linked to poverty (31). The shared load of housekeeping and close bonds between spouses boost the couple’s perception of efficacy in managing their houses, compensate for the scarcity of community resources, and protect against harmful community-level consequences (21). The other reason might be that the opposite effect of a woman’s greater wealth position may explain this occurrence since it makes her more financially autonomous of her partners, which would make it easier for her to exit an abusive relationship as well as less probable for her to support violence (29). Furthermore, wealthier status might also be associated with better formal educational achievement, which is likewise a factor in IPV; this may be particularly true in low- and middle-income nations (30).

The other factors which were associated with IPV were partner alcohol users. This association is in line with other studies conducted in Ethiopia (28, 32), Rwanda (33), and Nigeria (34). The probable reason for this association might be the effect of alcohol’s powerful behavioral influence. For instance, drinking too much alcohol might result in careless behavior, including poor judgment and difficulty understanding social rules, which raises the risk of IPV (34). Excessive alcohol consumption can also reduce family finances and increase conflict, both of which may contribute to IPV (35). The other reason could be alcohol’s direct effects on human physical and cognitive function, which impair self-control and make people less able to negotiate a peaceful settlement to disputes in associations, which could be used as a defense (28). According to the study, even if people have taken alcohol, those who think they have started acting more aggressively. This was demonstrated in experiments with both genuine and fake alcoholic beverages (33). The other effect might be that the partner used alcohol as a way to cope and reduce the stress and trauma that happened due to the conflict with their wife’s violence, even though the cause and effect are not determined in this study.

Another factor associated with IPV was the lack of formal partner education. This association is in concordance with other studies conducted in Ethiopia (11), Kenya (14), and East Africa (28). The possible reason for this association might be the effect of academics proposing that education is a means of fostering a sense of self-worth and enabling behavioral good (28). Education is a well-known informational resource and a strategy for fostering improvements in behavior. Therefore, ignorant spouses might not provide their wives independence, which is frequently motivated by cultural values (11).

## Strength and limitations

The utilization of a large sample size (72,672) of sub-Saharan African DHS makes this analysis more broadly applicable. There is adequate power to determine the true influence of the independent variables when using data from a large countrywide survey. Nevertheless, it is not without restrictions. For example, the cross-sectional structure of the data makes it impossible to discern a temporal relationship. Since the DHS dataset was secondary, it was not possible to figure out the data-specific measurement approach or parameters. The other weakness of this study is the effect of self-reported data on the highly sensitive issue of IPV.

## Conclusion and recommendation

The prevalence of IPV among pregnant women in sub-Saharan African countries was high (41.94%). The low economic status of participants, no formal education of the partner, and the partner’s alcohol consumption were associated with IPV. Enhancing their socioeconomic status and educational level while controlling their partner’s alcohol consumption is crucial for the prevention of IPV, based on the findings of this study. Therefore, this finding is crucial for policymakers and women’s rights protection to mitigate IPV. It is recommended that controlling the factors listed above is the best measure to prevent the occurrence of IPV. Future researchers are recommended to conduct advanced methods to show the causative relationship of IPV.

## Data Availability

The original contributions presented in the study are included in the article/supplementary materials, further inquiries can be directed to the corresponding author.
